# Acute Lower Respiratory Tract Infection Increased the Risk of Cardiovascular Events and All-Cause Mortality in Elderly Patients With Stable Coronary Artery Disease

**DOI:** 10.3389/fcvm.2021.711264

**Published:** 2021-09-17

**Authors:** Xiaoqian Zhao, Yuan Liu, Jinping Zhang, Shihui Fu, Chengyun Song, Yongyi Bai, Leiming Luo

**Affiliations:** ^1^Department of Cardiology, The Second Medical Center, National Clinical Research Center for Geriatric Diseases, Beijing Key Laboratory of Chronic Heart Failure Precision Medicine, Chinese PLA General Hospital, Beijing, China; ^2^Department of Cardiology, Chinese PLA 305 Hospital, Beijing, China; ^3^Department of Emergency, The Second Medical Center, National Clinical Research Center for Geriatric Diseases, Beijing Key Laboratory of Chronic Heart Failure Precision Medicine, Chinese PLA General Hospital, Beijing, China; ^4^Department of Cardiology, Hainan Hospital of Chinese PLA General Hospital, Beijing, China

**Keywords:** elderly, stable coronary artery disease, cardiovascular events, acute lower respiratory tract infection, all-cause mortality

## Abstract

**Objective:** To investigate the predictors of acute cardiovascular events within 90 days after an acute lower respiratory tract infection (ALRTI) in elderly patients with stable coronary artery disease (sCAD).

**Methods:** Observational analyses were conducted in a prospective cohort of the elderly with sCAD, during 90 days after they were hospitalized for ALRTI. Multiple logistic regression analysis was performed to identify predictors for acute cardiovascular events and all-cause mortality.

**Results:** The present study comprised 426 patients with sCAD (median age: 88 years; IQR: 84–91; range: 72–102). Among these patients, 257 suffering from ALRTI were enrolled in the infection group. Meanwhile, 169 patients who did not suffer from ALRTI were regarded as the non-infection group. Compared with the non-infection group, patients in the infection group had a higher incidence of acute cardiovascular events (31.9 vs. 13.6%, *p* < 0.001) and all-cause mortality (13.2 vs. 1.8%, *p* < 0.001) during the 90-day follow-up. In addition, in the infection group, the incidence of cardiovascular events was also higher than those in the non-infection group during the 7-day and 30-day follow-up (10.9 vs. 2.4%, *p* = 0.001; 20.6 vs. 6.5%, *p* < 0.001). The same difference in the incidence of all-cause mortality during 7 and 30 days (1.2 vs. 0%, *p* = 0.028; 3.9 vs. 0.6%, *p* = 0.021) was observed between the two groups. Furthermore, multiple regression analysis found that ALRTI was independently associated with increased risk of cardiovascular events and all-cause mortality in elderly patients with sCAD.

**Conclusion:** In elderly patients with sCAD, ALRTI was an independent predictor for both cardiovascular events and all-cause mortality.

## Introduction

Both acute lower respiratory tract infection (ALRTI) and coronary artery disease (CAD) occur more commonly in elderly individuals (1, 2). It is estimated that more than half of elderly patients with ALRTI who admitted to the hospital with this infection had a chronic cardiac condition (2). ALRTI is the most common infectious disease for hospitalization and a major cause for mortality in the elderly. Besides causing impairment and even failure for the respiratory system, ALRTI could also induce acute worsening of preexisting cardiovascular diseases or trigger new cardiovascular events (3–6).

There is an established link between cardiovascular events and the acute infection; the risk of adverse cardiovascular outcome is particularly augmented in the first few days after an acute infection, and might be sustained for several weeks (7–9). However, the magnitude of this problem has been underestimated for a long time, and until recently began to be appreciated fully. Among the very old patients with stable coronary artery disease (sCAD), there is a lack of enough evidence to link acute cardiovascular events (CVEs) to respiratory system infection, especially during the 90-day follow up after infection. In China, there are only few studies that focused on the relationship between cardiovascular complications and ALRTI, and the endpoints are limited in fast arrhythmias, transient ischemic attack (TIA), ischemic or hemorrhagic stroke, as well as venous thromboembolism (VTE).

The aims of the present study were to determine the incidence of various acute CVEs and all-cause mortality in a prospective cohort of elderly patients with sCAD after they were hospitalized for ALRTI and to investigate whether ALRTI is associated with a 90-day risk of CVEs and all-cause mortality in these patients.

## Methods

This was a single-center, prospective cohort study. This study was approved by the Ethics Committee of the Chinese PLA General Hospital and informed consent was obtained from all patients.

### Study Population

Based on the original adaptive study design and the pre-experiment results, allowing for a 20% dropout rate and statistical power of 80%, the planned sample size was 402. Originally, a total of 467 Chinese elderly patients (aged ≥60 years) with sCAD were enrolled consecutively from the Department of Geriatric Cardiology, Chinese PLA General Hospital between January 2011 and December 2013. Twenty-three patients were excluded because of unexplained death during the hospital, history of thromboembolism or disseminated intravascular coagulation recently, severe liver and kidney impairments, immune system disease, new onset or progression of malignant tumor, and having undergone radiotherapy and chemotherapy or surgery during last 30 days. In addition, 14 subjects were excluded because of data missing or loss of follow-up. Finally, 426 patients (median age 88 [IQR: 84–91; range: 72–102] years) were eligible for analysis. No significant differences were found in clinical characteristics between the included and excluded subjects.

### Data Management

The Chinese PLA General Hospital was the designated hospital of the included subjects and held their integrated long-term medical and final death records, which made it easier for us to follow them up effectively and judge endpoints accurately. Data were recorded in the database by trained doctors with full experience, and logistic check was performed by other doctors to ensure the accuracy of database. Only subjects with complete data were included in the final analysis.

All the patients with sCAD were given standard comprehensive treatments and did not accept percutaneous coronary intervention (PCI), coronary artery bypass graft (CABG), or other special treatments recently. A total of 257 patients with sCAD who suffered from ALRTI were enrolled in the infection group and accepted standard anti-infection therapy. Meanwhile, 169 sCAD patients who did not suffer from ALRTI were regarded as the non-infection group.

### Baseline Variables

After admission, the sociodemographic characteristics and clinical data were obtained from all participants as baseline variables. Clinical information included preexisting comorbidities, symptoms, signs, information of causative organisms and antibiotics for ALRTI, and variables of examinations and pertinent laboratory at admission. At various follow-up times, changes in laboratory tests, examination, CVEs, and deaths were collected and recorded.

sCAD diagnosis was based on 2013 European Society of Cardiology (ESC) guidelines on the management of sCAD (10). ALRTI diagnosis was referenced to 2011 guidelines for the management of adult lower respiratory tract infections (11).

Severity of ALRTI at presentation was quantified by means of the CURB-65 score, a validated predicting system for short-time mortality in patients with ALRTI. It calculates a score based on one demographic characteristic, three physical examination findings, and one laboratory test at the time of presentation. Based on this score, patients were stratified into three risk classes: low risk (0–1 points), moderate risk (2 points), and high risk (3–5 points). Each has an increasing risk of short-term mortality that ranged from 0.6% for lowest risk to 27.8% for highest risk (12).

In all patients, the estimated glomerular filtration rate (eGFR) was calculated using a Chinese modified version of the Modification of Diet in Renal Disease (MDRD) equation based on data from Chinese CKD patients as follows: 175 × serum creatinine (mg/dl)^−1.234^ × age (year)^−0.179^ × 0.79 (if female) (13). BMI was defined as the weight in kilograms divided by the square of the height in meters.

### Assessment of CVEs and Death

The incidences of six major CVEs, namely, acute ST elevation myocardial infarction (acute STEMI), no ST elevation acute coronary syndrome (NSTE-ACS), cerebral vascular accident (CVA), acute heart failure (AHF), new-onset atrial fibrillation (new-onset AF), and venous thromboembolism (VTE), were recorded. (1) Acute STEMI diagnosis was based on reported ST elevation on ECG along with positive plasma troponin test and a clinical diagnosis of STEMI (14). (2) NSTE-ACS was defined as acute non-ST elevation myocardial infarction [compatible ECG changes along with positive Troponin T result and a clinical diagnosis of non-STEMI or unstable angina (comprising chest pain with one of the ECG abnormalities, positive cardiac specific troponin test, or a clinical diagnosis of cardiac ischemia)] (14). An elevated cardiac specific troponin level might occur in ALRTI but without an acute coronary syndrome; hence, an elevated troponin level but without compatible clinical presentations was not regarded as an NSTE-ACS (15). (3) CVA included stroke and transient ischemia attack (TIA), stroke was defined as a new-onset neurological deficit lasting >24 h with confirmatory CT or MRI results. TIA was defined as a new-onset neurological deficit lasting <1 h without evidence of cerebral infarction (16). (4) AHF was defined as a new-onset or worsening heart failure that was detected by the managing physicians basing on the patient's symptoms, physical examination and laboratory tests, and documents in the medical record. (5) New-onset AF was symptomatic and diagnosed in patients without a prior history of AF; the two chief physicians made the diagnosis of AF for them based on clinical and ECG findings. (6) VTE, including pulmonary embolism (PE) and deep-vein thrombosis (DVT), was defined on the 2016 ACCP guideline and expert panel report for antithrombotic therapy for VTE disease (17). Information on death was followed up by communicating with the patient's family members at a fixed time and was then determined by reviewing the death record of patients.

### Definition of Basic Diseases

Hypertension was defined as systolic blood pressure≥140 mmHg and/or diastolic blood pressure ≥90 mmHg. The diagnosis was based on a medical history of hypertension, current treatment, and direct measurements performed on three different occasions during the study (18). Chronic heart failure was diagnosed by medical records, patient's symptoms, signs, and accessory examinations. Diabetes was defined as a history of diabetes, or fasting glucose >7 mmol/L, or 2-h postprandial blood glucose >11.1 mmol/L, or current treatment (18). Dyslipidemia was defined as a history of dyslipidemia or low-density lipoprotein cholesterol ≥3.36 mmol/L, high-density lipoprotein ≤1.03 mmol/L in males and ≤1.29 mmol/L in females, or current treatment (19). Chronic kidney disease (CKD) was determined as GFR <60 ml/min per 1.73 m^2^ based on KDOQI working group definition. Chronic obstructive pulmonary disease (COPD) was defined as treatment for asthma, chronic obstructive pulmonary disease, or the presence of interstitial lung disorders. Smoking was categorized according to tobacco consumption status in the previous year as current, former, or non-smoker.

### Follow-Up

The endpoints in this study were acute CVEs and all-cause mortality. All participants were followed up by telephone interviews or family visits at 7, 30, and 90 days after hospitalization, respectively. The endpoints were verified by reviewing hospital medical records. Death information was ascertained from the death records, a legal document including time, site, and other information.

### Statistical Analyses

Continuous variables were presented as mean ± standard deviation (SD) or median (with interquartile range); dichotomous variables were presented as numbers and percentages. Categorical variables were presented as counts, percentages, and incidence rate. Differences in baseline levels of risk factors and clinical characteristics between groups (infection or non-infection groups; patients with or without CVEs) were analyzed with chi-square or *t*-tests; the Wilcoxon two-sample test was used for continuous variables that were not normally distributed.

We used logistic regression models to identify predictors of CVEs. Potential predictors included demographic parameters (age, gender, comorbidities, and smoking history), clinical characteristics (physical examination, laboratory, and special examination items), and medications used, as listed in Tables 1, **3**. The cut-points for heart rate, SBP, DBP, and respiratory rate were based on results of a previous study (20), which explored factors affecting cardiovascular complications in patients with pneumonia.

**Table 1:** Characteristics of the infection and non-infection group at baseline.

**Variable**	**Infection Group**	**Non-infection Group**	***p*-value**
	**(*n* = 257), *n* (%)**	**(*n* = 169), *n* (%)**	
Age, years	88 (84, 91)	88 (85, 91)	0.37
Male	249 (96.9)	164 (96.5)	0.81
Smoking	105 (40.9)	55 (32.5)	0.08
Hypertension	192 (74.7)	149 (87.6)	<0.01
Diabetic	113 (44.0)	85 (50.0)	0.22
Dyslipidemia	123 (47.9)	89 (52.4)	0.36
CHF	53 (20.6)	26 (15.3)	0.17
AF	79 (30.7)	40 (23.5)	0.10
CKD	56 (21.8)	52 (30.6)	0.04
COPD	155 (60.3)	85 (50.0)	0.04
Anemia	76 (29.6)	40 (23.5)	<0.01
OMI	42 (24.9)	31 (12.1)	<0.01
Heart rate, bpm	82.7 ± 16.1	67.0 ± 10.0	<0.01
SBP, mmHg	131.4 ± 19.5	131.0 ± 18.7	0.83
DBP, mmHg	67.5 ± 11.1	68.1 ± 12.0	0.63
BMI, kg/m^2^	23.2 ± 3.4	24.5 ± 2.9	<0.01
LVEF, %	57.1 ± 6.9	59.4 ± 6.1	<0.01
LVEDD, mm	48.7 ± 5.6	49.4 ± 3.4	0.13
**Medication received**			
Anti-platelets	186 (72.4)	132 (78.1)	0.18
Statins	129 (50.2)	100 (59.2)	0.07
ACEIs or ARBs	114 (44.4)	96 (56.8)	0.08
β-blockers	136 (52.9)	99 (58.6)	0.25

*Values indicate median (interquartile range), mean (standard deviation), or percentage*.

*CHF, chronic heart failure; AF, atrial fibrillation; CKD, chronic kidney disease; COPD, chronic obstructive pulmonary disease; OMI, old myocardial infarction; bpm, beats per minute; SBP, systolic blood pressure; DBP, diastolic blood pressure; BMI, body mass index; LVEF, left ventricular ejection fraction; LVEDD, left ventricular end-diastolic diameter. ACEI, angiotensin-converting enzyme inhibitors; ARB, angiotensin II receptor blockers*.

In univariate analysis, we analyzed the relationship between potential factors and risk of events (CVEs and all-cause mortality) one by one without any adjustment. Factors with a significance level of *p* < 0.1 in the univariate analysis were entered into a forward stepwise multiple logistic regression analysis. Multivariate regression models were adjusted for age, sex, and other potential confounders (**Tables 4**, **5**). Results of logistic regression were reported as odds ratios (OR) and 95% confidence intervals (CI). The fulfillment of independence and collinearity were tested in the multivariate models. Interactive analysis was conducted to detect the association of between-factor interactions with CVEs and all-cause mortality. All statistical analyses were performed using SPSS (version 17.0, Inc., Chicago, IL, USA). A two-sided value of *p* < 0.05 was considered significant.

## Results

### Baseline Characteristics

The characteristics of the study population according to different groups (infection and non-infection groups) are summarized in Table 1. There were some significant differences between two groups, including history of hypertension, CKD, COPD, anemia, and old myocardial infarction (OMI), as well as heart rate, body mass index (BMI), and left ventricular ejection fraction (LVEF). Compared with the non-infection group, the infection group had higher heart rate and BMI and lower LVEF. The drugs used for sCAD were almost similar between the two groups, such as for anti-platelet agents, lipid-lowering agents, beta-blockers, and ACEI/ARB.

### Incidence of Acute CVEs and All-Cause Death in sCAD Patients With and Without ALRTI

During the 90-day follow-up, the infection group experienced 31.9% CVEs and 13.2% all-cause death, compared with 13.6 and 1.8% in the non-infection group, respectively (*p* < 0.01). In the cohort, during different periods of time after ALRTI, including 7, 30, and 90 days, all CVEs occurred in 32, 64, and 105 participants, respectively. The incidence of CVEs in the infection group was higher than those in the non-infection group, during the 7-day [28 (10.9%) vs. 4 (2.4%), *p* < 0.01)], 30-day [53 (20.6%) vs. 11 (6.5%), *p* < 0.01], and 90-day [82 (31.9%) vs. 23 (13.6%), *p* < 0.01] follow-up. All-cause mortality rates in the infection group were also higher than those in the non-infection group, during the 7-day [3 (1.2%) vs. 0 (0%), *p* = 0.28], 30-day [10 (3.9%) vs. 1 (0.6%), *p* = 0.06], and 90-day [34(13.2%) vs. 3(1.7%), *p* < 0.01] follow-up, respectively (Table 2). In addition, compared with the non-infection group, most of the CVEs and all-cause mortality in the infection group mainly occurred during the first 2 weeks after infection; those differences were sustained to 30 and 90 days after ALRTI.

**Table 2:** Occurrences of CVEs and death during the 90-day follow-up in the ALRTI and non-ALRTI group.

**Events**	**ALRTI group**	**Non-ALRTI group**	***p*-value**
	**(*n* = 257), *n* (%)**	**(*n* = 169), *n* (%)**	
**Combined CVEs**	82 (31.9)	23 (13.6)	<0.01
Acute STEMI	9 (3.5)	2 (1.2)	0.24
NSTE-ACS	40 (15.6)	16 (9.5)	0.07
CVA	11 (4.3)	2 (1.2)	0.07
AHF	4 (1.6)	0	0.16
New-onset Afi	12 (4.7)	0	<0.01
VTE	6 (2.3)	3 (1.8)	0.96
**All cause death**	34 (13.2)	3 (1.8)	<0.01

*ALRTI, acute lower respiratory tract infection; CVEs, cardiovascular events; acute STEMI, acute ST elevation myocardial infarction; NSTE-ACS, no ST elevation acute coronary syndrome; CVA, cerebral vascular accident; AHF, new-onset or worsening heart failure; new-onset Afi, new-onset atrial fibrillation; VTE, venous thromboembolism*.

### Comparison of Characteristics in Patients With and Without CVEs

According to the outcomes in this cohort, the participants were divided into two groups based on the occurrence or non-occurrence of acute CVEs, e.g., CVEs group with acute cardiovascular events and non-CVEs group without acute events during the 90-day follow-up. Compared with the non-CVEs group, the CVEs group had more histories of smoking (*p* < 0.05), CKD (*p* < 0.05), and COPD (*p* < 0.01). In the CVEs group, the respiratory rate (*p* < 0.001), heart rate (*p* < 0.05), plasma levels of blood urea nitrogen (BUN) (*p* < 0.05), fibrinogen (*p* < 0.01), C-reactive protein (CRP) (*p* < 0.05), and N-Terminal Pro-B type natriuretic peptide (NT-pro BNP) (*p* < 0.01) were significantly higher than those in the non-CVEs group (Table 3). Moreover, patients with ALRTI and high CURB-65 scores both had significantly increased cardiovascular events risks.

**Table 3:** Comparison of characteristics in patients with or without CVEs at baseline.

**Patient**	**CVEs group**	**No CVEs group**	***p*-value**
**characteristics**	**(*n* = 105), *n* (%)**	**(*n* = 321), *n* (%)**	
Age, years	88 (84, 92)	87 (83, 91)	0.85
Male	100 (95.2)	312 (97.2)	0.51
Smoking	29 (27.6)	132 (41.0)	0.01
BMI, kg/m^2^	23.3 ± 3.2	24.0 ± 3.2	0.07
Hypertension	82 (78.1)	259 (80.4)	0.60
Diabetic	51 (48.6)	147 (45.7)	0.60
Dyslipidemia	49 (46.7)	163 (50.6)	0.48
Chronic heart failure	17 (16.2)	62 (19.3)	0.48
Atrial fibrillation	26 (24.8)	93 (28.9)	0.41
Chronic kidney disease	36 (34.3)	72 (22.4)	0.02
COPD	47 (44.8)	193 (59.9)	0.01
Anemia	41 (39.0)	112 (34.8)	0.43
OMI	21 (20.0)	51 (15.9)	0.33
Respiratory rate ≥24 Breaths/min Breaths/min breathementagh	13 (12.4)	9 (2.8)	<0.01
breaths/min			
Heart rate ≥125 bpm	14 (13.3)	20 (6.2)	0.02
SBP <90 mmHg or DBP <60 mmHg	18 (17.1)	47 (14.6)	0.53
mmHg			
Plasma BUN, mmol/L	10.0 ± 5.2	8.7 ± 4.1	0.02
Plasma fibrinogen, g/L	4.2 ± 1.3	3.8 ± 1.1	<0.01
CRP, mg/dl	3.8 ± 5.4	2.7 ± 3.6	0.04
NT-proBNP, pg/ml	1,290 (342, 3,598)	439 (193, 1,713)	<0.01
LVEF, %	55.9 ± 8.2	55.8 ± 5.9	<0.01
ALRTI	82 (78.1)	175 (54.5)	<0.01
CURB-65, score	1.5 ± 1.0	1.0 ± 1.1	<0.01

*Values indicate median (interquartile range), mean (standard deviation), or percentage*.

*CVEs, cardiovascular events; BMI, body mass index; COPD, chronic obstructive pulmonary disease; OMI, old myocardial infarction; tpm, times per minute; bpm, beats per minute; SBP, systolic blood pressure; DBP, diastolic blood pressure; BUN, blood urea nitrogen; CRP, C-reactive protein; NT-proBNP, N end of b-type natriuretic peptide precursor; LVEF, left ventricular ejection fraction; ALRTI, acute lower respiratory tract infection*.

### Risk Factors Associated With CVEs

The univariate analysis revealed that risk factors associated with cardiovascular events were history of CKD, respiratory rate over 24 breaths/min, heart rate greater than 125 beats/min, and ALRTI, high CUR-65 scores, high plasma level of BUN, fibrinogen, CRP, and LVEF. In multivariate logistic regression models, independent predictors for CVEs were history of CKD (OR, 2.57 95% CI, 1.36–4.84, *p* < 0.01), ALRTI (OR, 2.67; 95% CI, 1.36–5.42, *p* < 0.01), and respiratory rate over 24 breaths/min (OR, 6.20; 95% CI, 1.73–22.2, *p* < 0.01; Table 4). In the interactive analysis, no between-factor interactions were found to be associated with CVEs (Supplementary Table 1).

**Table 4:** Risk factors associated with CVEs in elderly patients with SCAD.

	**Univariate**		**Multivariate^a^**	
	**OR (95% CI)**	***p*-value**	**OR (95% CI)**	***p*-value**
CKD	1.50 (0.92–2.45)	0.05	2.57 (1.36–4.84)	<0.01
ALRTI	2.99 (1.79–4.99)	<0.01	2.67 (1.36–5.24)	<0.01
CUR-65	1.51 (1.22–1.86)	<0.01		
Respiratory rate ≥24 breaths/min	10.23 (3.22–32.46)	<0.01	6.20 (1.73–22.25)	<0.01
Heart rate ≥125 bpm	4.69 (0.77–28.47)	0.04		
Plasma BUN	1.06 (1.02–1.12)	0.01		
Plasma Fib	1.34 (1.10–1.63)	<0.01		
CRP	1.06 (1.00–1.12)	0.05		
LVEF	0.94 (0.91–0.97)	<0.01		

a *Adjusted for age, sex, smoking, body mass index, hypertension, diabetic, chronic heart failure, atrial fibrillation, chronic kidney disease, chronic obstructive pulmonary disease, anemia, old myocardial infarction, respiratory rate ≥24 breaths/min; heart rate ≥125 bpm; systolic blood pressure <90 mmHg or diastolic blood pressure <60 mmHg; blood urea nitrogen; fibrinogen; C-reactive protein; N end of b-type natriuretic peptide precursor; left ventricular ejection fraction; acute lower respiratory tract infection; CURB-65*.

*CVEs, cardiovascular events; SCAD, stable coronary artery disease; OR, indicates odds ratio; CI, confidence interval; CKD, chronic kidney disease; ALRTI, acute lower respiratory tract infection; BUN, blood urea nitrogen; Fib, fibrinogen; CRP, C-reactive protein; LVEF, left ventricular ejection fraction*.

*Incident cardiovascular complications included any of the following cardiovascular events: Acute elevation myocardial infarction, no ST elevation acute coronary syndrome (acute non-ST elevation myocardial infarction or unstable angina), CVA, AHF, new-onset Afi, and VTE. Acute STEMI, acute ST elevation myocardial infarction; NSTE-ACS, included NSTEMI and UA; cerebral vascular accident; new or worsening heart failure; new-onset atrial fibrillation; venous thromboembolism (pulmonary embolism and deep-vein thrombosis)*.

### Risk Factors Associated With All-Cause Mortality

There were 10 factors that had significant associations with all-cause mortality in the univariate analysis model [age, CKD, ALRTI, CUR-65 score, BMI, breaths greater than 24 bpm; plasma levels of BUN, albumin (ALB), and triglycerides (TC)]. Among them, only three variables remained independently associated with all cause death during the 90-day follow up in our multivariate model: ALRTI (OR, 3.53; 95% CI, 1.02–12.3, *p* = 0.047), plasma levels of BUN (OR, 1.11; 95% CI, 1.03–1.21, *p* = 0.01), and prealbumin (OR, 0.92; 95% CI, 0.86–0.89, *p* = 0.02). The magnitude and significance of these associations are presented in Table 5. In the interactive analysis, no between-factor interactions were found to be associated with all-cause mortality (Supplementary Table 2).

**Table 5:** Risk factors associated with all-cause mortality in elderly patients with SCAD.

	**Univariate**		**Multivariate^a^**	
	**OR (95% CI)**	***p*-value**	**OR (95% CI)**	***p*-value**
Age	1.09 (1.01–1.17)	0.06		
ALRTI	8.47 (2.56–28.0)	<0.01	3.53 (1.02–12.3)	0.047
CUR-65	2.44 (1.69–3.53)	<0.01		
BMI	0.79 (0.68–0.91)	<0.01		
Respiratory rate ≥24 breaths/min	3.81 (1.16–12.5)	0.03		
Hb	0.97 (0.95–0.99)	0.01		
Tlc	0.001 (0.00–0.06)	<0.01		
Plasma BUN	1.09 (1.02–1.16)	0.01	1.11 (1.03–1.21)	0.01
Serum Mg	0.01 (0.00–0.18)	<0.01		
Serum TC	0.44 (0.21–0.94)	0.03		
Serum ALB	0.90 (0.83–0.97)	<0.01		
Serum PA	0.91 (0.86–0.97)	<0.01	0.92 (0.86–0.98)	0.02

a *Adjusted for age, sex, smoking, body mass index, hypertension, diabetic, chronic heart failure, atrial fibrillation, chronic kidney disease, chronic obstructive pulmonary disease, anemia, old myocardial infarction, respiratory rate ≥24 breaths/min; heart rate ≥125 bpm; systolic blood pressure <90 mmHg or diastolic blood pressure <60 mmHg; white blood cell; hemoglobin; total lymphocyte count; thrombocyte; alanine transaminase; aspartate transaminase; blood urea nitrogen; creatinine; serum glucose; triglycerides; high-density lipoprotein cholesterol; low-density lipoprotein cholesterol; serum sodium; potassium; calcium; magnesium; phosphorus; albumin; prealbumin; fibrinogen; C-reactive protein; N end of b-type natriuretic peptide precursor; left ventricular ejection fraction; acute lower respiratory tract infection; CURB-65*.

*SCAD, stable coronary artery disease; OR, odds ratio; CI, confidence interval; ALITI, acute lower respiratory tract infection; BMI, body mass index; Hb, hemoglobin; Tlc, total lymphocyte count; BUN, blood urea nitrogen; TC, total cholesterol; ALB, serum albumin; Serum PA, serum prealbumin*.

In addition, relationship among the independent predictors was investigated. Respiratory rate, plasma BUN, and serum PA were found to be significantly associated with ALRTI in elderly patients with SCAD (Supplementary Table 3).

## Discussion

The main finding of the present study was that ALRTI increased the incidence of CVEs in elderly patients with sCAD. These events tend to be particularly common in the early course of ALRTI.

Among all events, the most frequent was NSTE-ACS; however, the incidence of STEMI was lower than that reported by previous studies. Musher and colleagues (21, 22) reported that the incidence of STEMI was 7.1% at the time of admission in 170 patients hospitalized for bacterial pneumonia caused by *Streptococcus pneumoniae*, which was higher than that in the present study. It might be due to the difference in the investigated subjects. In Musher's report (22), the incidence of new-onset or worsening congestive heart failure and new-onset arrhythmia (atrial flutter, atrial fibrillation, and ventricular tachycardia with the exclusion of terminal arrhythmias) after respiratory system infection were 14 and 5.8%, respectively, higher than our results of 0.7 and 2.3%. Potential explanations for the disparity are that the definition of new-onset or worsening congestive heart failure is based on the different diagnostic criteria. In line with previous studies, we also found that the risk of CVEs would sustain for 90 days after infection. Meanwhile, a similar association of ALRTI with CVEs was found in patients admitted with ALRTI.

In the present study, we found that ALRTI caused various CVEs. ALRTI is considered to be an acute and systematic condition, which not only affected the lungs, but also involved other systems and organs, such as the cardiovascular system and heart. Platelet activation plays a pivotal role in orchestrating inflammatory events that trigger, or more likely exacerbate, vascular damage and dysfunction, supported by the steadily increasing recognition of the critical role played by these cells in mediating inflammatory and immune responses (23–26). Bacteria and their products activate and aggregate the platelets. Circulating inflammatory mediators or direct infection of cardiomyocytes with pneumonia-causing organisms, or both these mechanisms, might result in an impairment of vessel endothelium, balance disorder of coagulation and fibrinolysis system, water and sodium retention, myocardial ischemia and energy metabolism disorder, cardiac electrical activity abnormity, and so on (27), which may be responsible for various acute CVEs by causing ischemic, hemorrhagic, heart failure, and arrhythmic events. Moreover, it was found that there was a sustained influence of ALRTI on adverse outcomes for at least 90 days, which meant that the impairments were not temporary but extended to several months.

The main strength of our study is that patients with well-documented medical history served as the study participants. The subjects of the present study belonged to the same medical facility and socioeconomic status and were cared for by the same physicians. The Chinese PLA General Hospital was their designated hospital and had the integrated long-term medical and final death records of patients, which made it easier for us to follow-up effectively and judge endpoints accurately. The ascertainment of ALRTI was based on prespecified clinically relevant definitions and was systematically carried out for all patients on the basis of electronic clinical records, with the aim of reducing information bias.

The present study has potential limitations. First, the participants investigated was from a single military hospital, the number was relatively small, and most of the subjects were very old male. Many of the subjects had multiple risk factors for coronary artery disease, so there is the possibility of residual confounding resulting in the likelihood of underestimating CVEs. In addition, the single-center design and the composition of research objects may partially affect the extrapolation of the observations of the present study. Finally, we failed to find out the related risk factors of various CVEs. Several studies have shown that associated arrhythmias may also be a risk factor for the development of a TIA or ischemic stroke. The coagulopathy associated with severe pneumonia may also be a potential risk factor for cerebral hemorrhage (24).

However, studies on the adverse outcomes in very old people with sCAD after ALRTI were sparse in China, especially in global CVEs and all-cause death, so this finding would benefit the prevention for adverse outcomes in the elderly. In view of the high incidence of CVEs and adverse outcomes in Chinese elderly ALRTI patients with sCAD, early intervention for preventing various CVEs would be given, such as anti-coagulation therapy, strictly controlling water and sodium retention, and myocardium protection.

In conclusion, in elderly patients with sCAD, ALRTI increased the risk of CVEs and all-cause mortality during the 90-day follow-up. The acute lower respiratory tract infection was an independent predictor for both short-term CVEs and all-cause mortality. Further related measurements should be made for those high-risk patients with clustering of risk factors. The effects of commonly used cardiovascular drugs on the course of respiratory infection and the effects of common antibiotics on the cardiovascular systemic balance should be investigated further.

## Data Availability Statement

The raw data supporting the conclusions of this article will be made available by the authors, without undue reservation.

## Ethics Statement

The studies involving human participants were reviewed and approved by the Ethics Committee of the Chinese PLA General Hospital. The patients/participants provided their written informed consent to participate in this study. Written informed consent was obtained from the individual(s) for the publication of any potentially identifiable images or data included in this article.

## Author Contributions

YB, LL, and JZ conceptualization and funding acquisition. XZ, YL, and YB methodology. SF and CS formal analysis. XZ and JZ data curation. XZ and YB writing—original draft preparation. All authors have read and agreed to this version of the manuscript.

## Funding

This work was supported by grants from Clinical Research of Chinese PLA 305 hospital (17YQ08), the Open Project of the National Clinical Research Center for Geriatric Diseases (NCRCG-PLAGH-2019025), and the Medical Big Data Project of PLA General Hospital (2019-MBD-020).

## Conflict of Interest

The authors declare that the research was conducted in the absence of any commercial or financial relationships that could be construed as a potential conflict of interest.

## Publisher's Note

All claims expressed in this article are solely those of the authors and do not necessarily represent those of their affiliated organizations, or those of the publisher, the editors and the reviewers. Any product that may be evaluated in this article, or claim that may be made by its manufacturer, is not guaranteed or endorsed by the publisher.
