# Supplementary material for: Acute Lower Respiratory Tract Infection Increased the Risk of Cardiovascular Events and All-Cause Mortality in Elderly Patients With Stable Coronary Artery Disease
Source: Front Cardiovasc Med. 2021 Sep 17;8:711264. doi: 10.3389/fcvm.2021.711264 (PMC8484318; doi:10.3389/fcvm.2021.711264)
Supplement: Supplementary file 1 [file Data_Sheet_1.docx]

**Supplemental Table 1.** **The association of** **between-factor interactions with CVEs in elderly patients with SCAD**

|  | **OR (95%CI)** ^a^ | **P value** |
| --- | --- | --- |
| ALRTI*CKD | 1.56 (0.51-4.76) | 0.43 |
| ALRTI*Respiratory rate≥24 | 3.87(0.929-4.55) | 0.16 |
| CKD* Respiratory rate≥24 | 10.06(0.84-19.88) | 0.07 |

^a^ The association of between-factor interactions with CVEs was detected by logistic regression analysis. Abbreviations: CVEs, cardiovascular events; SCAD, stable coronary artery disease; OR, indicates odds ratio; CI, confidence interval; CKD, chronic kidney disease; ALRTI, acute lower respiratory tract infection.

**Supplemental Table 2. The association of between-factor interactions with all-cause mortality in elderly patients with SCAD**

|  | **OR (95%CI)** | **P value** |
| --- | --- | --- |
| ALRTI* Plasma BUN | 0.88 (0.65-1.18) | 0.39 |
| ALRTI* Serum PA | 0.94 (0.80-1.11) | 0.48 |
| Plasma BUN * Serum PA | 0.99 (0.98-1.01) | 0.99 |

^a^ The association of between-factor interactions with CVEs was detected by logistic regression analysis. Abbreviations: SCAD, stable coronary artery disease; OR, indicates odds ratio; CI, confidence interval; ALITI, acute lower respiratory tract infection; BUN, blood urea nitrogen; Serum PA: serum prealbumin.

**Supplemental Table 3. The association of** **ALRTI with other independent predictors**

|  | **OR (95%CI)** ^a^ | **P value** |
| --- | --- | --- |
| CKD | 1.082(0.713-1.640) | 0.712 |
| Respiratory rate≥24 | 4.811(1.079-21.445) | 0.039 |
| Plasma BUN | 1.066(1.015-1.120) | 0.011 |
| Serum PA | 0.915(0.881-0.951) | <0.001 |

^a^ The association of ALRTI with other independent predictors was detected by logistic regression analysis. Abbreviations: ALRTI, acute lower respiratory tract infection; CKD, chronic kidney disease; BUN, blood urea nitrogen; Serum PA: serum prealbumin.
